# Comparison of Salivary pH Changes after Consumption of Two Sweetened Malaysian Local Drinks among Individuals with Low Caries Experience: A Pilot Study

**DOI:** 10.21315/mjms2018.25.4.10

**Published:** 2018-08-30

**Authors:** Eswara Uma, Kan Sze Theng, Lynndy Lim Huan Yi, Low Hong Yun, Eby Varghese, Htoo Htoo Kyaw Soe

**Affiliations:** 1 Department of Pediatric Dentistry, Faculty of Dentistry, Melaka Manipal Medical College (MMMC), Manipal Academy of Higher Education (MAHE), Malaysia; 2 Tiew Dental Clinic, Petaling Jaya, Kuala Lumpur, Malaysia; 3 Dental Clinic Bandar Maharani, Muar, Johor, Malaysia; 4 Dental Clinic Yong Peng, Johor, Malaysia; 5 Research Methodology and Biostatistics Unit, Department of Community Medicine, Faculty of Medicine, Melaka Manipal Medical College (MMMC), Manipal Academy of Higher Education (MAHE), Malaysia

**Keywords:** saliva, pH, beverage, soft drinks

## Abstract

**Background:**

Regular consumption of sweetened beverages has been associated with dental caries, which is mediated through salivary pH. The salivary pH changes among individuals with low caries experience after consumption of two local sweetened drinks is compared.

**Methods:**

In this block randomised controlled parallel group, an open-label pilot study of 49 participants aged 21–25 were selected. The participants were randomised into three groups: mineral water, chocolate drink and *sirap bandung*. One day prior to the test, scaling was done and the participants did not eat or drink anything on the test day till the saliva sample collection was done. Salivary pH was measured at baseline and after the consumption of the drinks with a glass electrode digital pH meter at five-minute interval for half an hour. The statistical significance was assessed at the level of 5%.

**Results:**

After consumption of chocolate drink and *sirap bandung*, the salivary pH dropped from a baseline of 7.09 for chocolate drink and 7.13 for *sirap bandung* to 6.69 for chocolate and 6.86 for *sirap bandung*. This difference was statistically significant (*P* < 0.001).

**Conclusion:**

Sweetened milk based local drinks can increase the caries susceptibility. In the community, information about limiting the intake of sweetened drinks should be reinforced.

## Introduction

Diet and nutrition play a major multifactorial role in the etiology and pathogenesis of oral diseases. While the diet exerts a local effect on the oral health with respect to the integrity of teeth, salivary composition, pH and plaque formation, nutrition has a systemic effect (1). Sugars, in the form of carbohydrates, are the principal components of an individual’s diet and classified as “intrinsic sugars” (naturally integrated into the cellular structure of food; for example, fruits) and “extrinsic sugars” (occur as free molecules or are added to the food). Milk sugars along with non-milk extrinsic sugar (NMES), which include fruit juices, honey and added sugars especially during manufacture, in recipe sugars and in table sugars, are extrinsic sugar sub-group. Interestingly, foods high in NMES are the ones consumed frequently; for example, sweetened beverages between meals (2). Some studies showed an increased role of refined sugars, such as sucrose, an extrinsic sugar, in causing diseases ranging from dental caries to delinquent behaviour as well unhealthy lifestyle, especially in young adults (2–4).

Since Miller’s chemoparasitic theory that described the cause of dental caries, several modifying factors have been identified that render a complex etiology for the dental caries model. The modifying factors include saliva, the immune system of the body, time, socio-economic status, education level of the individual, lifestyle behaviours and the use of fluorides (1). Saliva plays a major role in maintaining oral health. Alteration of the oral homeostasis that is maintained by saliva, leads to poor oral health (5). This is primarily because saliva performs multiple roles within the oral cavity. The buffering capacity of saliva helps in neutralising the acids produced from the plaque, foods and beverages. As saliva is saturated with calcium and phosphate ions and is in equilibrium with enamel, the enamel dissolution does not occur. When the pH of the saliva drops below the critical pH, the enamel dissolves to restore the calcium and phosphate ion equilibrium to normal (6). Thus, the salivary buffer is the key to preventing dental erosion and caries (4).

In Malaysia, increasing availability and consumption of sugars and sweeteners coupled with increasing sedentary lifestyles have contributed to the rising problems of obesity and associated non-communicable diseases (3). Frequent consumption of beverages sweetened with condensed milk, chocolate, cordial syrup and sugars by adults have contributed significantly towards energy intake (3, 7, 8). In addition to the aerated beverages available in the market, Malaysians frequently consume many locally prepared beverages like *teh tarik*, *sirap bandung* and *soya cincau* as they are prepared at the food stalls itself and sold at a very affordable price. Due to the affordability and ease of availability, these beverages are all-time favourite drinks among the locals. Some of these beverages are also perceived to be healthy as they have milk as the main ingredient as opposed to the aerated beverages.

Previous studies have shown an irreversible effect of fruit juices and aerated beverages on the teeth (9–11), while milk exerts a protective effect (12) that is attributed to its remarkably lower fermentation by oral bacteria as compared to that of sucrose. In addition, the fermentation product of lactose in milk, lactic acid, inhibits the growth of several pathogenic microbes (13). Although the literature describes the effects of aerated beverages and juices on the salivary and plaque pH, the effect of locally prepared and popular milk-based sweetened drinks such as *sirap bandung* and chocolate drink, both containing NMES (14), have not yet been elucidated.

Despite several preventive measures instituted by the government, the prevalence of dental caries in Malaysia among 20–24 years old is 81.9% (15). Among Malaysians, the frequent consumption of these sweetened beverages may be one of the contributing factors to the high prevalence of dental caries. Thus, the present study aimed to assess the changes in the pH of saliva after the consumption of two sweetened Malaysian drinks among individuals with low caries.

## Materials and Methods

### Participants

This randomised, controlled, parallel group, open-label, pilot study was conducted in our institution. The ethical clearance from the institute was obtained from the Human and Ethics committee prior to the start of the study (Ref# - MMMC/FOD/AR/B3/E C-2015(03)). Using purposive sampling, dental students were invited to participate in the study. A total of 146 students volunteered to participate in the study. The study procedure was explained to the participants and informed consent obtained before starting the study. In addition, the history of each participant regarding any acute or chronic disease of the body or oral cavity as well as the use of any antibiotics two months before the collection of saliva samples was collected by interview. The history of any deleterious habits like smoking or chewing tobacco was also obtained. A single examiner using a mouth mirror and explorer examined all the subjects. The teeth were cleaned with cotton, dried using an air syringe, and examined for the presence of caries. The tooth surface was probed only if the visual examination was inconclusive. The decayed, missing and filled teeth (DMFT) were recorded based on the WHO criteria (16). Oral Hygiene Index–Simplified (OHI-S) comprising of debris index and calculus index was recorded for all the participants (17). Six key surfaces (four from posterior teeth and two from anterior teeth) were examined for debris index and calculus index. Buccal surfaces of teeth #16 and #26 and lingual surfaces of teeth #36 and #46 were scored. In the anterior region, the labial surfaces of teeth #11 and #31 were scored. The OHI-S score was calculated after computing the debris and calculus scores.

The participants of the study included dental students of the institution aged 21–25 years. Only those students with DMFT < 3 and OHI-S < 1.2 were included in the study. Students undergoing orthodontic treatment (fixed or removable) and having any systemic illness or a history of intake of any antibiotics or medications that could affect the salivary function or pH, within two months before the collection of saliva samples, were excluded from the study (18, 19).

A literature search did not retrieve any study that was conducted using the same sweetened drinks as the ones used in the present pilot study. Using the guidelines for sample size estimation for a pilot study as well as a similar previous study, a minimum sample size of 15 participants per sweetened group was planned for the present pilot study (20, 21).

**Figure 1:**
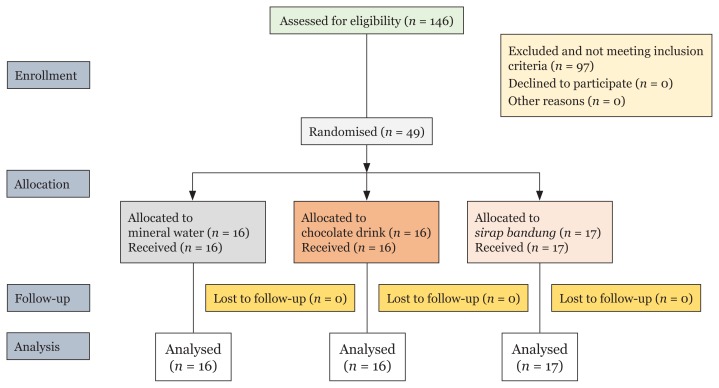
Flow chart of participant allocation

Of the 146 voluntary participants, 49 dental students (32 females and 17 males) with a mean age of 23.3 ± 0.77 years were eligible to participate in the study after applying the inclusion and exclusion criteria. The participants were randomly assigned to one of the three groups, namely mineral water (B1), chocolate drink (B2) and *sirap bandung* (B3) in 1:1:1 ratio using research randomiser. Mineral water and chocolate drink groups consisted of 16 participants each, and *sirap bandung* group had 17 participants (Figure 1). Each selected participant was assigned a code used during saliva collection.

### Test Products

Three beverages were selected for the study: mineral water (B1)-Spritzer®, chocolate drink (B2)-Milo® powder, and *sirap bandung* (B3)-rose syrup (F&N) in milk. These products were selected only by their popularity and not sponsored by any of the companies of the products involved. The sweetened beverages were prepared fresh in the morning on the day of the study using a standard recipe with the modification of excluding the ice (22, 23). For preparing the chocolate drink, six tablespoons of Milo powder were used with two tablespoons of condensed milk (F&N) and dissolved in 500 mL water. For preparing *sirap bandung*, three cups of sugar and three tablespoons of evaporated milk (F&N) were dissolved in 750 mL water with rose water (F&N) added as a flavouring. The intrinsic pH of the test beverage was measured after the drink was prepared freshly.

### Digital pH Meter

In the present study, we used a digital pH meter (glass combination electrode-Mettler Toledo™ FE20) that was initially calibrated using three standard buffer solutions of pH 4.0, 7.0 and 10.0. A two-point calibration using standardised buffer solutions (pH 2 and pH 10) was conducted daily prior to the first analysis. The participants drooled the saliva sample in a 15 mL sterile test tube. The saliva collected at the 5 min interval was at least 2 cm from the bottom of the test tube excluding the froth on the top. This ensured that the glass bulb of the electrode sensor was completely immersed in the saliva sample to obtain an accurate and stable reading that was recorded from the display of the pH meter. After each measurement, the glass electrode was cleansed carefully with deionised water and blotted dry using filter paper before the next sample was analysed. The protocol was repeated for each saliva sample.

In order to reduce the bias and maintain the high accuracy of results, each examiner was trained for calibration in the chemistry laboratory of the institution. This procedure included a theoretical overview related to saliva, its collection, and the determination of its pH. We also included the discussion of issues that might be encountered during the study period.

### Experimental Procedure

The subjects of each group underwent scaling and polishing one day before the test and were instructed to refrain from eating in the morning on the day of the study till the saliva sample was collected. The salivary pH can be affected by the individual factors, such as flow rate and buffering capacity, and extrinsic factors, such as the frequency and time of the day of the consumption of food/beverage, volume of the beverage consumed, duration of intake of a particular beverage, as well as, the temperature at which the beverage was consumed. The method of dispensing the beverage to each group and the saliva collection method were standardised to account for the confounding factors. A volume of 200 mL of the room temperature beverage was dispensed to each participant. To account for the circadian rhythm, the beverages were given at the same time, i.e., 10 a.m., on the test day. The beverage was given only one time, and the participants were asked to consume it within 5 min of the baseline pH measurement of the saliva.

On the day of the test, at the given time, the subjects of a specific group chewed on paraffin wax while seated in a relaxed position. Then, the stimulated saliva was collected into a sterile test tube for 5 min. The pH of the stimulated saliva was recorded as the baseline pH value. Subsequently, the group was given 200 mL of the room temperature beverage. The salivary sample of each subject was collected in separate sterile test tubes, coded with a specific identity number assigned to each subject prior to the study, at 5, 10, 15, 20 and 30 min intervals after beverage consumption. The changes in the pH were measured immediately with the pre-calibrated portable digital pH meter in combination with a glass electrode.

**Figure 2:**
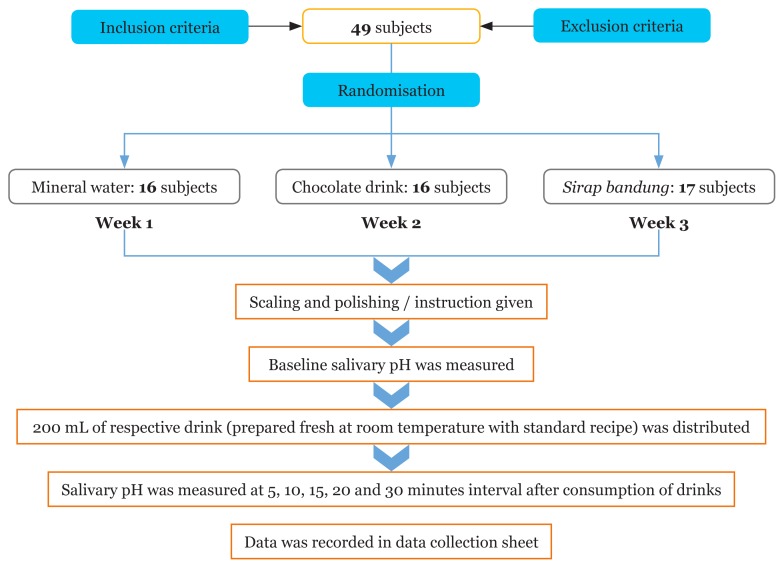
Flow chart showing method of the study

The present study was conducted over a period of three weeks, with one group tested in one week (Figure 2).

## Statistical Analysis

Data were analysed using SPSS version 18. Descriptive statistics, such as mean and standard deviation, were calculated. Paired *t*-test was used to compare the pH of saliva at different time points with baseline using ANOVA followed by Bonferroni adjustment between the three groups. The statistical significance was assessed at *P* < 0.05.

## Results

A total of 49 healthy subjects were randomly administered one of the three beverages such as mineral water, chocolate drink and *sirap bandung*. The descriptive statistics of the study are shown in Table 1. The intrinsic pH values of all the three beverages assessed in this study are stated in Table 2. Consequently, *sirap bandung* was found to be slightly more acidic with an intrinsic pH of 6.17 as compared to mineral water and chocolate drink with an intrinsic pH of 7.02 and 7.0, respectively.

**Figure 3:**
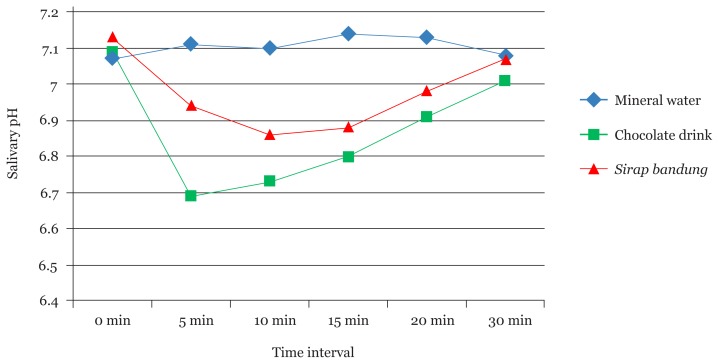
Comparison of salivary pH changes between mineral water, chocolate drink and *sirap bandung*

The changes in salivary pH after consumption of beverages as compared to baseline pH in the three groups are shown in Table 3 and Figure 3. In the mineral water group, no significant difference was observed in the salivary pH at 5 (*P* = 0.353), 10 (*P* = 0.388), 15 (*P* = 0.108), 20 (*P* = 0.163) and 30 (*P* = 0.845) min as compared to baseline pH. However, salivary pH was significantly reduced at 5 (*P* < 0.001), 10 (*P* < 0.001), 15 (*P* < 0.001) and 20 (*P* < 0.01) min as compared to the baseline salivary pH in the chocolate drink and *sirap bandung* groups. At 30 min, no significant difference was detected in the salivary pH in the chocolate drink (*P* = 0.074) and *sirap band*ung (*P* = 0.172) as compared to the baseline pH.

The comparison of changes in the salivary pH among mineral water, chocolate drink, and *sirap bandung* group is shown in Table 4. Furthermore, no significant difference was detected in the salivary pH between mineral water, chocolate drink and *sirap bandung*. Moreover, the salivary pH was significantly different at 5 (*P* < 0.001), 10 (*P* < 0.001), 15 (*P* < 0.001) and 20 (*P* = 0.014) min among the three beverages but not at 30 min (*P* = 0.552). It was also observed that while the pH returned to baseline value in mineral water group at 30 min, same was not achieved in the chocolate and *sirap bandung* groups. The changes in the salivary pH were compared between the beverages to identify the one causing a significant change (Table 5). At baseline, no significant difference was detected in the salivary pH between the three groups. At 5 min, the chocolate drink had a significantly lower salivary pH (*P* < 0.001) but not *sirap bandung* as compared to mineral water. Furthermore, both the chocolate drink and *sirap bandung* showed a significant drop in the salivary pH at 10 and 15 min. At 20 min, the pH was significantly lower in the chocolate drink as compared to the mineral water (*P* = 0.013) but not in *sirap bandung* (*P* = 0.141). However, no statistically significant change was observed in the salivary pH between chocolate drink and *sirap bandung*.

## Discussion

The present study reported the changes in salivary pH after consumption of sweetened popular Malaysian drinks. Several studies (18, 21, 24, 25) have been conducted to evaluate the changes in salivary pH after consumption of fruit juices and soft drinks, while only a few studies reported the effect of locally prepared popular beverages on the salivary pH. In order to study this effect, we conducted a pilot study.

In the current study, the randomisation of eligible participants, ensured a near uniform distribution across the three groups based on gender and ethnicity as 23 years old were selected for the study as dental caries prevailed in this age group in Malaysia (15). Moreover, the behaviour of an individual and cultural and social influences served as the determinants of caries and caries risk (1). This cohort of participants has also been exposed to fluorides from childhood (1) and has grown up drinking the locally prepared sweetened beverages. To study the effects of sweetened drinks in individuals with low caries experience, participants having low caries experience with DMFT < 3 (16) were selected for this study. Reportedly, among Malaysians, the greatest contributor to daily sugar intake was self-prepared drinks, such as tea, coffee, cordial syrups, chocolate drinks and fruit juices, to which sugar was added (3). This information aided in choosing the sweetened drinks for the study: chocolate drink and cordial drink (*sirap bandung*). Studying the effects of changes in salivary pH in individuals after consuming the popular sweetened local beverages can help in educating the community.

Previous studies investigated the effect of fruit juices and soft drinks with respect to the changes in plaque pH; whereas, in the present study, salivary pH was evaluated as the participants were undergraduate dentistry students, who were conscious about oral hygiene. In order to conduct a plaque pH study, the participants had to refrain from tooth brushing for 48 h, and in the case of our participants, it would have been difficult to ascertain if they adhered to the restriction.

Mineral water and chocolate drink were found to be near neutral pH (7.02 and 7.0, respectively), while *sirap bandung* was slightly below neutral with a pH of 6.17. Both the sweetened drinks, irrespective of their intrinsic pH, caused a significant drop in salivary pH. These results were consistent with the changes observed after consumption of any sweetened beverage (18, 24). This phenomenon might be attributed to the rapid metabolism of the sugars in the beverage by acidogenic bacteria in the residual plaque, tongue, mucosa and saliva (26). The properties of saliva, the type of sugars in the beverage, their concentration and the baseline pH largely influence the changes in the observed salivary pH (27).

The difference observed in the salivary pH between the sweetened drinks and mineral water was that while the consumption of sweetened drinks led to a drop in salivary pH, the consumption of mineral water led to a slight increase in the pH that was sustained significantly up to 20 min. This finding was similar to that reported by Azrak et al. An increase in the salivary pH was attributed to the low buffering capacity of the mineral water and probably to the gustatory stimulus post-consumption (27).

The chocolate drink caused a significant drop in the salivary pH at 5 min interval as compared to *sirap bandung*. The salivary pH recovered in 20 min although it was still significantly lower than the baseline pH. *Sirap bandung* caused a significant drop in the salivary pH up to 10 min, following which, the pH recovered although it was significantly lower than the baseline pH. The current findings are parallel to the changes in the pH of saliva observed when mineral water was compared to the orange juice, whole milk and fennel tea during the period of 5–10 min (27). Chocolate drink and *sirap bandung* are highly cariogenic due to the high sugar content. The chocolate drink contained roughly one and a half tablespoons of sugar, while *sirap bandung* contained two and a half tablespoons of sugar per 200 mL of the drink. The sucrose in the beverages is easily fermented into lactic acid and pyruvic acid that are responsible for the drop in the salivary pH post-consumption. The initial decline in the pH was due to the speed at which the aciduric and acidogenic bacteria in the mouth could metabolise sucrose. Simultaneously, the enzymatic activity of glucosyltransferase is increased leading to the formation of glucan polymer essential for the adherence of *Streptococcus mutans* to the teeth and bacterial aggregation. With repeated consumption, the plaque thickness accelerates, causing an acidic environment of the teeth that cannot be overcome easily by the buffers in saliva, thereby increasing the risk of caries in an individual (24). Both sweetened beverages contain milk solids. Milk and milk derivatives are generally known to contain caries protective components, such as calcium, phosphorus, glycoproteins, proteoglycans and lactophorins. Whole milk is accepted as a non-cariogenic beverage due to its lactose content (2). However, the anti-cariogenic properties of the milk are lost when the milk-containing beverages are sweetened (13, 27, 28).

Chocolate drink and *sirap bandung* are sweetened drinks that are regularly consumed by the local population. The drop in the salivary pH after consumption of the test drinks was significant when prepared and consumed at room temperature; usually, these beverages are consumed when chilled. Thus, the temperature of the drinks might affect the acidity of the drinks because freezing changes the physical state (from liquid to a solid phase) of the beverages as the solute concentrates (the molecules are tightly packed) and the buffering capacity decreases leading to a prolonged decline in the pH. The consumption of cold drinks can cause a marked pH fall, thereby necessitating more than usual volume of alkaline salivary buffering action to rinse and normalise the pH of the oral environment (18). The results of the previous studies demonstrated the effect of temperature of the drink on salivary pH; thus, if the test beverages were to be consumed chilled as opposed to room temperature, the salivary pH would drop further.

In this pilot study, the salivary pH after consumption of the sweetened beverages did not fall below the critical pH as described previously. The critical pH for the dissolution of enamel in saliva was critical for the salivary concentration of calcium and phosphates. In individuals with low salivary concentrations of calcium and phosphate, the critical pH maybe 6.5, while the critical pH in individuals with higher concentrations of salivary calcium and phosphate maybe 5.5, indicating that critical pH is not a constant (6). The individual variation in salivary concentration of calcium and phosphate and its effect on the critical pH might be attributed to the salivary pH not falling below the critical pH in the groups. In addition, the drop in the salivary pH is not solely substrate-driven; other variables also influence the pH drop. The primary factors being the buffering capacity of saliva as well as the salivary flow rate vary among the individuals, which in turn, could lead to diverse results in contrast to when the same individuals are given different test beverages (21). Widowati et al. showed that the saliva pH in patients with high caries experience decreased markedly as compared to patients with low caries experience after consuming the snacks containing sucrose and maltitol (24). The participants in this study had DMFT < 3, and therefore, did not have salivary pH declining close to the critical pH. It should be highlighted that individuals with multiple caries and active lesions might experience a drastic fall in the salivary pH posing a huge risk for the rapid progression of dental caries if they consume these beverages frequently and regularly as the ability of the saliva to neutralise the acid decreases (21, 24).

Thus, these results indicated that when the oral cavity is subjected to a sweetened substrate challenge, salivary pH levels fall. Soft drinks and fruit juices are acidic and known to have severe erosive potential (29). Herein, the sweetened milk-based drinks tested are cariogenic due to their ability to cause a significant drop in the salivary pH. Therefore, excessive and abusive consumption of these sweetened beverages can have detrimental effects on the teeth. As dental professionals, it becomes mandatory for us to educate the community about the use of these popular local sweetened beverages through dental health education and diet counseling (18).

## Conclusion

This study suggested that the milk-based chocolate drink and *sirap bandung* cause a significant drop in the salivary pH in individuals with low caries experience and exposure to fluoride. The “2013 Malaysian dietary guidelines” recommended choosing plain water instead of sugary drinks and limiting the intake of table sugar or sweetened condensed milk and sweetened beverages, such as cordial and carbonated drinks. These practices should be reiterated and reinforced in the community (30).

## Figures and Tables

**Table 1:** Participant demographics (*n* = 49)

Variables	Mineral water (*n* = 16) *n* (%)	Chocolate drink (*n* = 16) *n* (%)	*Bandung* (*n* = 17) *n* (%)
Age (years)*	23.7 (0.7)	23.2 (0.7)	22.9 (0.9)
Sex			
Male	5 (31.3)	6 (37.5)	6 (35.3)
Female	11 (68.7)	10 (62.5)	11 (64.7)
Race			
Malay	8 (50.0)	4 (25.0)	9 (52.9)
Chinese	7 (43.7)	8 (50.0)	5 (29.4)
Indian	1 (6.3)	4 (25.0)	3 (17.7)

* Mean (SD)

**Table 2:** The intrinsic pH of all three beverages

Beverage	Code	pH
Mineral water	B1	7.02
Chocolate drink	B2	7.00
Sirap Bandung	B3	6.17

**Table 3:** Change in salivary pH after consumption of beverages compared to baseline pH

Time interval	Mineral water ( B1)				Chocolate drink (B2)				*Sirap bandung* (B3)			
		
	pH values^a^	Mean diff. (95% CI)	*t*-statistic (df)	*P*-value^b^	pH values^a^	Mean diff. (95% CI)	*t*-statistic (df)	*P*-value^b^	pH values^a^	Mean diff. (95% CI)	*t*-statistic (df)	*P*- value^b^
Baseline	7.07 (0.16)				7.09 (0.26)				7.13 (0.15)			
5 min	7.11 (0.16)	−0.04 (−0.11, 0.04)	0.95 (15)	0.353	6.69 (0.40)	0.4 (0.2, 0.5)	4.9 (15)	< 0.001	6.94 (0.16)	0.2 (0.1, 0.2)	7.2 (16)	< 0.001
10 min	7.10 (0.18)	−0.03 (−0.1, 0.04)	0.8 (15)	0.388	6.73 (0.27)	0.4 (0.2, 0.5)	6.4 (15)	< 0.001	6.86 (0.18)	0.3 (0.2, 0.4)	6.9 (16)	< 0.001
15 min	7.14 (0.21)	−0.07 (−0.15, 0.02)	1.7 (15)	0.108	6.80 (0.21)	0.3 (0.2, 0.4)	4.9 (15)	< 0.001	6.88 (0.18)	0.2 (0.2, 0.3)	5.6 (16)	< 0.001
20 min	7.13 (0.18)	−0.06 (−0.2, 0.02)	1.5 (15)	0.163	6.91 (0.24)	0.2 (0.1, 0.3)	3.2 (15)	0.005	6.98 (0.21)	0.1 (0.1, 0.2)	3.6 (16)	0.003
30 min	7.08 (0.16)	−0.01 (−0.1, 0.1)	0.2 (15)	0.845	7.01 (0.21)	0.1 ( 0.01, 0.1)	1.9 (15)	0.074	7.07 (0.19)	0.1 (−0.03, 0.2)	1.4 (16)	0.172

a mean (SD)

b Paired *t*-test

**Table 4:** Salivary pH comparison between mineral water, chocolate drink and *sirap bandung*

pH value	Mineral water^a^ mean (SD)	Chocolate drink^a^ mean (SD)	*Sirap bandung*^a^ mean (SD)	F( df_1_,df_2_)	*P*-value^b^
Baseline	7.07 (0.16)	7.09 (0.26)	7.13 (0.15)	0.45 (2, 46)	0.640
5 min	7.11 (0.16)	6.69 (0.40)	6.94 (0.16)	10.34 (2, 46)	< 0.001
10 min	7.10 (0.18)	6.73 (0.27)	6.86 (0.18)	12.54 (2, 46)	< 0.001
15 min	7.14 (0.21)	6.80 (0.21)	6.88 (0.18)	12.30 (2, 46)	< 0.001
20 min	7.13 (0.18)	6.91 (0.24)	6.98 (0.21)	4.65 (2, 46)	0.014
30 min	7.08 (0.16)	7.01 (0.21)	7.07 (0.19)	0.60 (2, 46)	0.552

a mean (SD)

b ANOVA

**Table 5:** Comparison of salivary pH between mineral water and chocolate drink; mineral water and *sirap bandung*; chocolate drink and *sirap bandung*

Intervals (minutes)	Comparison of mineral water versus chocolate drink			Comparison of mineral water versus *sirap bandung*			Comparison of chocolate drink versus *sirap bandung*		
		
	Sal. pH of mineral water^a^	Sal. pH of chocolate drink^a^	*P*–value^b^	Sal. pH of mineral water^a^	Sal. pH of *sirap bandung*^a^	*P*–value^b^	Sal. pH of chocolate drink^a^	Sal. pH of *sirap bandung*^a^	*P*–value^b^
Baseline	7.07 (0.16)	7.09 (0.26)	0.999	7.07 (0.16)	7.13 (0.15)	0.999	7.09 (0.26)	7.13 (0.15)	0.999
5	7.11 (0.16)	6.69 (0.40)	< 0.001	7.11 (0.16)	6.94 (0.16)	0.205	6.69 (0.40)	6.94 (0.16)	0.027
10	7.10 (0.18)	6.73 (0.27)	< 0.001	7.10 (0.18)	6.86 (0.18)	0.007	6.73 (0.27)	6.86 (0.18)	0.246
15	7.14 (0.21)	6.80 (0.21)	< 0.001	7.14 (0.21)	6.88 (0.18)	0.002	6.80 (0.21)	6.88 (0.18)	0.700
20	7.13 (0.18)	6.91 (0.24)	0.013	7.13 (0.18)	6.98 (0.21)	0.141	6.91 (0.24)	6.98 (0.21)	0.981
30	7.08 (0.16)	7.01 (0.21)	0.945	7.08 (0.16)	7.07 (0.19)	0.999	7.01 (0.21)	7.07 (0.19)	0.999

a mean (SD)

b ANOVA with Bonferroni adjustment
